# Genome-Wide Search for Competing Endogenous RNAs Responsible for the Effects Induced by Ebola Virus Replication and Transcription Using a trVLP System

**DOI:** 10.3389/fcimb.2017.00479

**Published:** 2017-11-21

**Authors:** Zhong-Yi Wang, Zhen-Dong Guo, Jia-Ming Li, Zong-Zheng Zhao, Ying-Ying Fu, Chun-Mao Zhang, Yi Zhang, Li-Na Liu, Jun Qian, Lin-Na Liu

**Affiliations:** ^1^Academy of Military Medical Sciences, Beijing, China; ^2^Key Laboratory of Jilin Province for Zoonosis Prevention and Control, Changchun, China

**Keywords:** competing endogenous RNA network, ebola virus, cellular response changes, circRNA, microRNA, lncRNA

## Abstract

Understanding how infected cells respond to Ebola virus (EBOV) and how this response changes during the process of viral replication and transcription are very important for establishing effective antiviral strategies. In this study, we conducted a genome-wide screen to identify long non-coding RNAs (lncRNAs), circular RNAs (circRNAs), micro RNAs (miRNAs), and mRNAs differentially expressed during replication and transcription using a tetracistronic transcription and replication-competent virus-like particle (trVLP) system that models the life cycle of EBOV in 293T cells. To characterize the expression patterns of these differentially expressed RNAs, we performed a series cluster analysis, and up- or down-regulated genes were selected to establish a gene co-expression network. Competing endogenous RNA (ceRNA) networks based on the RNAs responsible for the effects induced by EBOV replication and transcription in human cells, including circRNAs, lncRNAs, miRNAs, and mRNAs, were constructed for the first time. Based on these networks, the interaction details of circRNA-chr19 were explored. Our results demonstrated that circRNA-chr19 targeting miR-30b-3p regulated CLDN18 expression by functioning as a ceRNA. These findings may have important implications for further studies of the mechanisms of EBOV replication and transcription. These RNAs potentially have important functions and may be promising targets for EBOV therapy.

## Introduction

Ebola virus (EBOV) causes a severe hemorrhagic fever, and EBOV-related experiments must be performed under biosafety level (BSL) 4 conditions. However, there are very few laboratories owning BSL-4 conditions and this may limit the study of EBOV. Our study sought to determine the competing endogenous RNAs (ceRNAs) that impact the virulence of EBOV using a transcription and replication-competent virus-like particle (trVLP) system that models the life cycle of EBOV under BSL 2 conditions (Hoenen et al., 2014). The tetracistronic minigenome of trVLPs contains the EBOV leader region, luciferase reporter, VP40, GP, VP24, EBOV trailer region and three non- coding regions from the EBOV genome that are essential to produce trVLPs containing these minigenomes. After pre-transfection with EBOV NP, VP35, VP30, and L expression vectors, the cells can be infected by the trVLPs. Thus, the trVLP system can be used as a tool to explore the cellular mechanism changes related to EBOV replication, transcription, morphogenesis, budding, and entry (Hoenen et al., 2014). The trVLP system was used to perform a rapid screening assay of effective viral polymerase inhibitors of EBOV, and the anti-EBOV activity of the selected drugs was confirmed using fully infectious Zaire EBOV, indicating that this system can be used for studies of virus replication and the development of novel antivirals (McCarthy et al., 2016; Biedenkopf and Hoenen, 2017).

To establish effective antiviral strategies, it is necessary to understand how cells respond to infection and how this response changes during the course of viral replication and transcription (Holzer et al., 2016). In the past decades, RNA research related to EBOV has mainly included miRNA and mRNA. In 2014, a series of miRNAs were identified as novel targets for Ebola drugs (Liang et al., 2014; Sheng et al., 2014; Yan and Gao, 2014). In 2015, Yue Teng and Yuzhuo Wang conducted a genome-wide screen and predicted miRNAs in EBOV, suggesting two possible EBOV-encoded miRNAs might be silenced, resulting in the down-regulation of the target genes NFKBIE and RIPK1 (Teng et al., 2015). In 2016, a novel study presented evidence of EBOV-induced changes in circulating miRNA populations in both nonhuman primates (NHPs) and humans, indicating these miRNAs could be exploited as pathogen-specific diagnostic biomarkers (Duy et al., 2016). In addition, the *in silico* inhibition of EBOV by anti-Ebola miRNAs has been achieved (Golkar et al., 2016). One study identified an EBOV-encoded miR-155 homolog that may regulate importin-a5 expression and facilitate evasion of the host immune system (Liu et al., 2016). A report examining host gene expression presented an overview of differentially expressed genes in human and bat cells infected with EBOV or Marburg virus and demonstrated more rapid filovirus replication in human cells than in bat cells (Holzer et al., 2016).

Recently, pseudogenes, long non-coding RNAs (lncRNAs), and circular RNAs (circRNAs) were found to act as miRNA “sponges” that affect miRNA activity (Ebert and Sharp, 2010; Salmena et al., 2011; Cesana and Daley, 2013). These ceRNAs, including circRNAs and ncRNAs, may play important roles in many human diseases, such as Alzheimer's disease, pulmonary fibrosis, human breast cancer and many other cancer types (Song et al., 2014; Chiu et al., 2015; Conte et al., 2017; Wang et al., 2017). However, the ceRNAs responsible for the effects induced by EBOV replication and transcription have not been reported.

**Figure 1:**
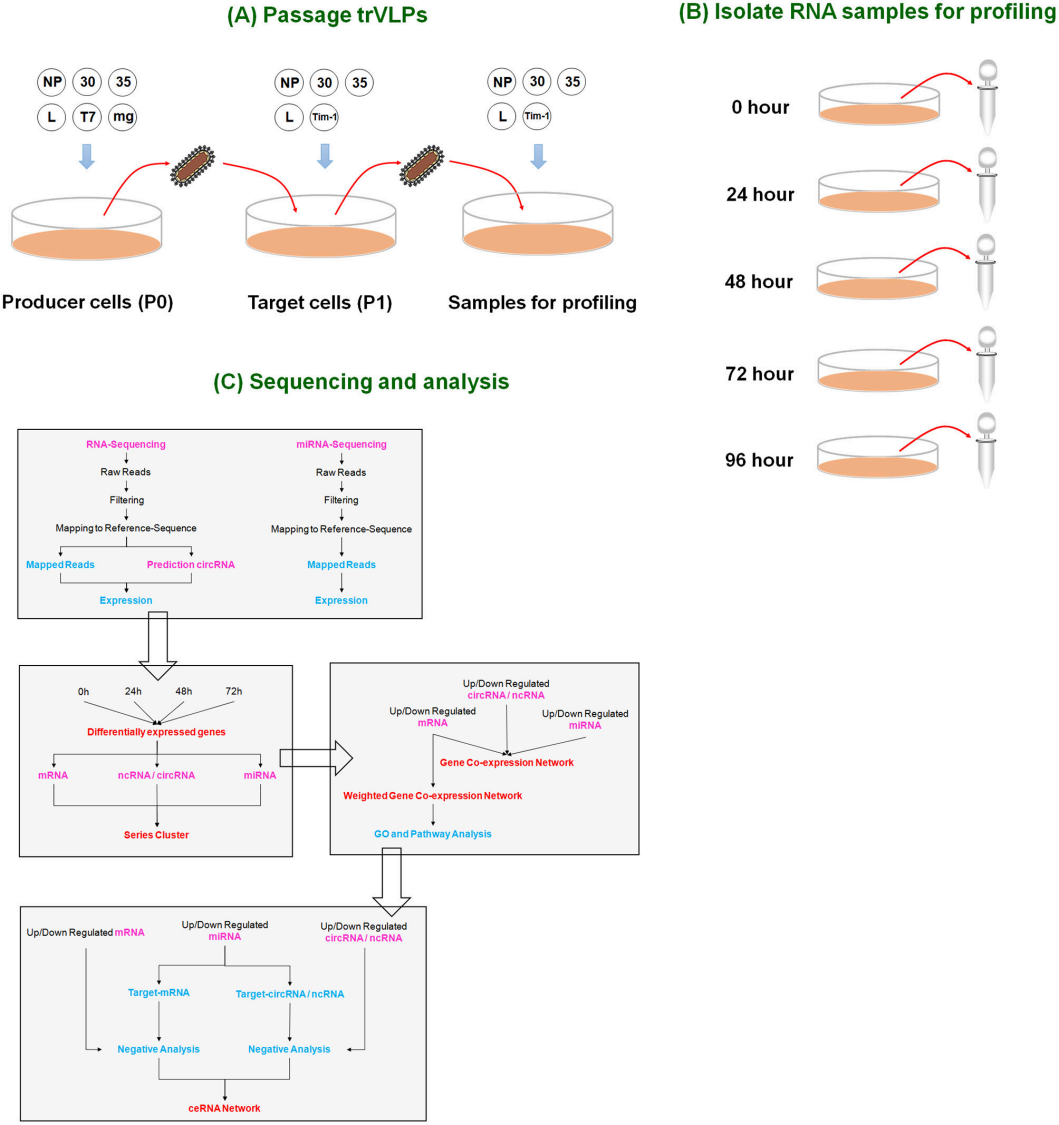
Overview of the experimental design. **(A)** Passage of trVLPs. P0 trVLPs were obtained from producer cells containing plasmids (NP, 30, 35, L, T7, and minigenome). P0 trVLPs were then used to infect target cells to obtain P1 trVLPs. The P1 trVLPs were used to prepare samples for profiling. **(B)** Isolation of RNA samples for profiling. P1 trVLPs were used to infect target cells in a 6-well plate (2 ml/well). The P1 trVLP TCID_50_ was 10^−6.5^/ml, and there were 4 × 10^5^ infected cells/well. Five time points were selected to harvest samples for sequencing based on trVLP infection and replication (see Figure 2). **(C)** Profiling and analysis. Genome-wide RNA sequencing and miRNA sequencing were performed to identify the ceRNAs responsible for the effects induced by Ebola viral infection and replication in human cells.

In this study, we conducted a genome-wide search for lncRNAs, circRNAs, miRNAs, and mRNAs and identified those that were differentially expressed during viral replication and transcription using a tetracistronic trVLP system that models the life cycle of EBOV in 293T cells (Figure 1). We next performed a series cluster analysis to identify the expression patterns of these differentially expressed RNAs. Up- or down-regulated genes were selected to establish a gene co-expression network based on co-expression patterns. CeRNA networks were constructed based on target analysis and gene expression negative analysis. We chose miR-30b-3p and verified its interactions with related circRNAs and mRNAs.

## Materials and methods

### Sample preparation

We use pCAGGS-L, pCAGGS-NP, pCAGGS-VP30, pCAGGS-VP35, and pCAGGS-T7 for the initial production of trVLPs, and pCAGGS-L, pCAGGS-NP, pCAGGS-VP30 pCAGGS-VP35, and pCAGGS-Tim1 were used to prepare target cells. The details of trVLP production and infection were as described previously (Hoenen et al., 2014). P1 trVLPs were used to infect 293T cells, which were pre-transfected with pCAGGS-L, pCAGGS-NP, pCAGGS-VP30, pCAGGS-VP35, and pCAGGS-Tim1. The TCID_50_ of P1 trVLPs was 10^−6.5^/ml. Approximately 4 × 10^5^ cells were present in each well of a 6-well plate, and the P1 trVLPs used for infection in this experiment were applied in a volume of 2 ml per well. There were three wells per time point for the next analysis steps. Each well was considered an independent experiment. Total RNA was extracted from cells using TRIzol reagent (Invitrogen). RNA quality was checked using a Bioanalyzer 2200 (Aligent), and RNA was stored at −80°C. miRNA was purified using a miRNeasy Mini Kit (Qiagen) and validated by performing gel electrophoresis.

### Luciferase assay for trVLP reproduction

Renilla luciferase was detected using a Renilla-Glo Assay Kit (Promega). A 40-μl aliquot of each sample was added to 40 μl of Renilla-Glo reagent, and the light produced was measured in a luminometer using an integration time of 1 s.

### TCID_50_ assay

TCID_50_ assays of p1 trVLPs were performed in 96-well tissue culture plates. The pre-transfection volume of plasmids per well in a 96-well plate was 1/10 of the volume per well in the 6-well plate. The p1 trVLPs were diluted to different concentrations. The cell culture medium was removed from the cells before the addition of p1 trVLPs. Then, 100 μl of diluted p1 trVLPs was added to each well of the 96-well plate. After 24 h, the p1 trVLP inoculum was replaced by common cell culture medium. The reporter activity was detected 72 h post infection by the method previously described. Wells with RLU values three times higher than that of the control group were identified as positive. TCID_50_ was then calculated by the Karber method.

### Western blots

Infected 293T cells were lysed with lysis buffer for protein extraction. A bicinchoninic acid (BCA) protein assay kit (Pierce) was used to determine the protein concentration. Proteins were separated by sodium dodecyl sulfate–polyacrylamide gel electrophoresis (10%) and were electro-transferred to a nitrocellulose blotting membrane (GE Healthcare Life Science) using a Trans-Blot SD Semi-Dry Transfer Cell (Bio-Rad) for 25 min at room temperature. Blots were incubated overnight at 4°C with antibodies against EBOV-GP and EBOV-VP40 (1:2,000 dilution; horse antibody, provided by Professor Yang Songtao) (Zheng et al., 2016), followed by incubation for 1 h at room temperature with a horseradish peroxidase-conjugated rabbit anti-horse secondary antibody (Bioss Inc., Beijing). Proteins were detected by chemiluminescence (Millipore), and β-actin was used as an internal control. The primary antibody for the internal control was mouse β-actin monoclonal antibody (Santa Cruz Biotechnology), and the secondary antibody was donkey anti-mouse IgG (H+L) highly cross-adsorbed secondary antibody, Alexa Fluor 680 (Thermo). Fluorescent detection was performed using the Odyssey infrared imaging system (Li-cor Biosciences). CLDN18 was detected by rabbit anti-CLDN18 antibody (Abcam), and the secondary antibody was goat anti-rabbit IgG HRP-linked antibody (Cell Signaling). The secondary antibody used to detect the internal control for CLDN18, β-actin, was peroxidase-conjugated goat anti-mouse IgG antibody (Biosharp).

### Quality control

Raw sequencing data were tested by performing FAST-QC, and the evaluation metrics included quality distribution of nucleotides, position-specific sequencing quality, GC content, the proportion of PCR duplication, kmer frequency, and others.

### cDNA library construction

cDNA libraries were constructed for each pooled RNA sample using VAHTSTM Total RNA-seq (H/M/R). In general, the protocol consisted of the following steps: the RNA was depleted of rRNA and fragmented into 150–200 bp by incubation with divalent cations at 94°C for 8 min. The cleaved RNA fragments were reverse-transcribed into first-strand cDNA, followed by second-strand cDNA synthesis, end repair, A-tailing and ligation with indexed adapters. The target bands were harvested using VAHTSTM DNA Clean Beads. The products were purified and enriched by PCR to create the final cDNA libraries and quantified by an Agilent2200. The tagged cDNA libraries were pooled at equal ratios and used for 150-bp paired-end sequencing in a single lane of the Illumina Xten with 51 plus 7 cycles.

### miRNA library construction and RNA sequencing

The complementary DNA (cDNA) libraries for single-end sequencing were prepared using the Ion Total RNA-Seq Kit v2.0 (Life Technologies). The cDNA library was size selected by PAGE gel electrophoresis for miRNA sequencing. The cDNA libraries were then processed for proton sequencing. After diluting and mixing the samples, the mixture was processed on a OneTouch 2 instrument (Life Technologies) and enriched on a OneTouch 2 ES station (Life Technologies) to prepare the template-positive Ion PI™ Ion Sphere™ Particles (Life Technologies) according to the Ion PI™ Template OT2 200 Kit v2.0 (Life Technologies). After enrichment, the mixed template-positive Ion PI™ Ion Sphere™ particles in the samples were loaded onto a P1v2 Proton Chip (Life Technologies) and sequenced on a proton sequencer according to the Ion PI Sequencing 200 Kit v2.0 (Life Technologies).

### Gene expression calculation

Reads per Kb per million reads (RPKM) was used to calculate gene expression from RNA-seq data. RPKM indicates the expression level of a specific gene and eliminates the influence of gene length and sequencing amount when calculating gene expression, facilitating the direct comparison of differentially expressed genes among different samples. If multiple transcripts existed for a gene, we selected the longest one to calculate sequencing depth and expression.

### Series cluster

We identified a number of model expression patterns for different types of RNAs. The raw expression values were converted into log_2_ ratios. Some unique profiles were defined by employing a strategy to cluster short time-series gene expression data. The expression model profiles showed relationships with the actual or expected numbers of genes involved in each model profile. Significant profiles had higher probabilities than expected using Fisher's exact test and a multiple comparison test (Miller et al., 2002; Ramoni et al., 2002).

### Co-expression network analysis

Gene co-expression network analyses were performed to reveal the relationships between differentially expressed genes. The co-expression networks were constructed by calculating Pearson correlations. We used k-core scores to search the core regulatory genes that had the highest networking degrees in the networks (Kim et al., 2001; Ravasz et al., 2002; Prieto et al., 2008).

### CeRNA network construction

lncRNAs, circRNAs, and mRNAs with expression levels that shared meaningful correlations were subjected to ceRNA analysis. We searched for potential miRNA response elements (MREs) among the lncRNA, circRNA, and mRNA sequences, and overlaps between the same miRNA seed sequence binding sites in both lncRNA/circRNA and mRNA sequences were considered predictive of lncRNA/circRNA-miRNA-mRNA interactions. miRNA binding sites were predicted using miRbase (http://www.mircode.org/), and miRNA–mRNA/miRNA–lncRNA/miRNA–circRNA interactions were predicted using miRanda (Pasquinelli, 2012).

### Real-time PCR

Total RNA was isolated from 293T cells using TRIzol reagent and then reverse-transcribed using random primers. RNA expression levels are reported relative to glyceraldehyde 3-phosphate dehydrogenase (GAPDH). All steps were performed according to the manufacturer's instructions (Applied Biosystems, USA). The primers used in the present study were as follows:

GAPDH Forward: CACCATCTTCCAGGAGCGAG,
GAPDH Reverse: GCAAATGAGCCCCAGCCT;
CircRNA-chr19 Forward: GGCATAGTCACAGTGCGGAC,
CircRNA-chr19 Reverse: CAGCCTTTAACGGACTTGCA.
CLDN18 Forward: ACATGCTGGTGACTAACTTCTG
CLDN18 Reverse: AAATGTGTACCTGGTCTGAACAG

### Luciferase reporter analysis

The CLDN18 3′-UTR containing the miR-30b-3p binding site and a mutation of this site were inserted into pmiR-RB-ReportTM. 293T cells were transfected with wild-type or mutated reporter vectors, miRNA expression vectors, and a negative control. Lysates were harvested 48 h after transfection. Renilla luciferase activities were detected according to the dual-luciferase assay manual (Promega).

### RNA interference and transfection

293T cells were transfected with siRNAs using Lipofectamine 2000 (Invitrogen) according to the manufacturer's protocol.

siRNA-ceRNA-chr19-1 sense:
    GACUUGUGUGGUGAGAAACTT
siRNA-ceRNA-chr19-1 antisense:
    GUUUCUCACCACACAAGUCTT
siRNA-ceRNA-chr19-2 sense:
    GUGGUGAGAAACCCUACAATT
siRNA-ceRNA-chr19-2 antisense:
    UUGUAGGGUUUCUCACCACTT

## Results

### Characterization of a trVLP system modeling the life cycle of EBOV

The trVLPs contain a minigenome that comprises the leader and trailer non-coding regions, VP24, VP40 and GP genes of the EBOV genome as well as a luciferase reporter gene. These trVLPs infect target cells, replicate, and transcribe genes encoding the L, VP35, VP30, and NP proteins, which are cloned into expression plasmids (Figure 1). Reporter activity in target cells is an indicator of trVLP replication and transcription. The trVLPs can be continuously passaged to model multiple infectious cycles.

**Figure 2:**
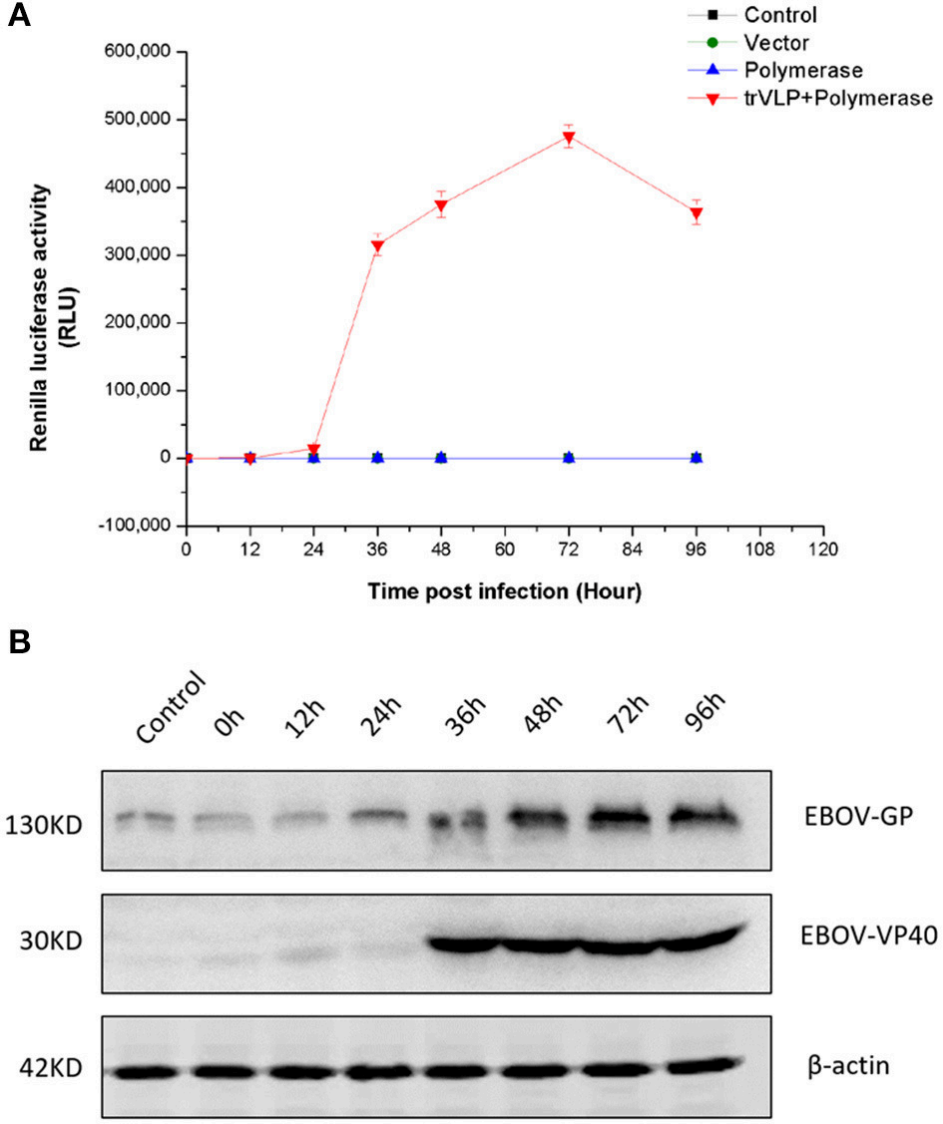
Tetracistronic trVLP production in 293T cells. **(A)** Tetracistronic trVLP replication curve based on the detection of luciferase activity. In this experiment, P1 virus-like particles were used to infect target 293T cells (P1 cells) that had been transfected with helper plasmids (pCAGGS-NP, pCAGGS-VP35, pCAGGS-L, pCAGGS-VP30, pCAGGS-Tim-1) 1 day prior to infection, and there were three control groups (Control: transfected with only Transit LT1; Vector: transfected with empty pCAGGS; and Polymerase: transfected with pCAGGS-NP, pCAGGS-VP35, pCAGGS-L, pCAGGS-VP30, pCAGGS-Tim-1). Cell lysates were diluted 1:100 prior to the detection of luciferase activity, and each time point was measured in triplicate. The TCID_50_ of P1 trVLPs was 10^−6.5^/ml, and the inoculation volume was 2 ml/well in 6-well plates (4 × 10^5^ cells/well). The results represent the mean ± SD of 3 different preparations. **(B)** Detection of viral protein expression. To investigate the expression of EBOV-GP and EBOV-VP40 by western blotting, we used a polyclonal antibody produced in horse against EBOV-GP or EBOV-VP40.

Renilla signal levels reflect minigenome replication and transcription and indicate trVLP production and entry into P1 cells. Renilla signals began to increase sharply 24 h after infection and reached a peak at 72 h after infection. Thereafter, signal levels at 96 h decreased to the levels observed at 48 h (Figure 2A). Western blotting revealed a similar expression pattern for EBOV-GP and an obviously different expression pattern for EBOV-VP40 in P1 cells compared to controls (Figure 2B).

Thus, we successfully constructed a system to produce replication-competent trVLPs that infect and reproduce in 293T cells utilizing several assistance measures (as described above). Based on our results, trVLP-infected cells may be used to a certain extent in lieu of EBOV-infected host cell samples for transcriptome sequencing to investigate ceRNAs related to viral replication and transcription. We repeated this experiment in 293T cells and selected samples in triplicate at 24, 48, 72, and 96 h for RNA extraction and sequencing (Figure 1B).

### RNA expression profile throughout the EBOV life cycle

**Figure 3:**
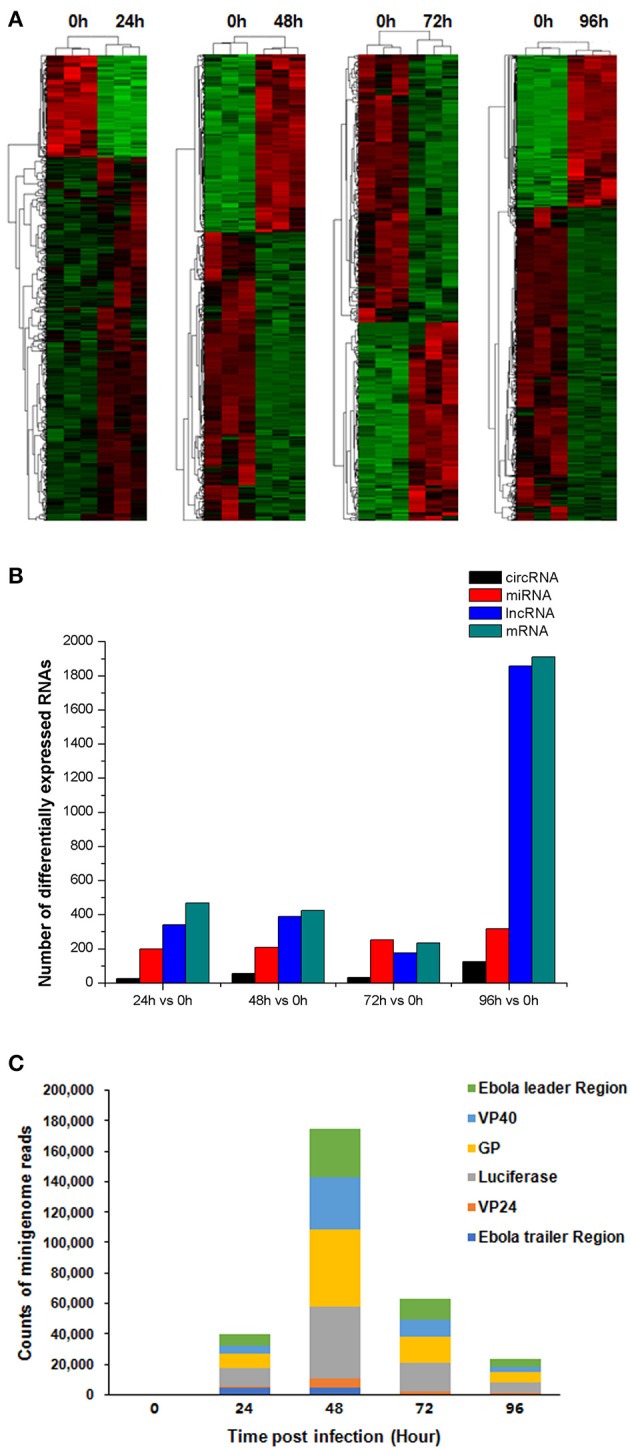
RNA expression profile and hierarchical clustering analysis. **(A)** Hierarchical clustering analysis. The Limma algorithm was applied to filter differentially expressed lncRNAs, circRNAs, mRNAs and miRNAs at four time points (24, 48, 72, and 96 h) compared with the 0-h time point post virus infection (*P* < 0.05, fold change > 2). **(B)** Differentially expressed RNA numbers. The numbers of differentially expressed circRNAs, lncRNAs, miRNAs, and mRNAs are indicated at four time points compared with the 0-h time point. **(C)** Minigenome read numbers. We searched the vRNA sequences of trVLPs in a partial profile that was not mapped to the human genome. The reads at five time points included the following sequences: Ebola leader region, VP40, GP, VP24, Ebola trailer region and Renilla luciferase. Read numbers at each time point represent the averages of triplicate samples.

We obtained an expression profile comprising 51,136 genes, which constituted a global expression signature. We then analyzed lncRNAs, circRNAs, mRNAs, and miRNAs that were differentially expressed at 4 time points (24, 48, 72, and 96 h post virus infection) compared with their expression at the 0-h time point (Figure 3A). We identified 342 lncRNAs, 26 circRNAs, 468 mRNAs and 199 miRNAs that were differentially expressed between 24 and 0 h; 389 lncRNAs, 56 circRNAs, 425 mRNAs and 209 miRNAs between 48 and 0 h; 177 lncRNAs, 33 circRNAs, 233 mRNAs, and 252 miRNAs between 72 and 0 h; and 1,858 lncRNAs, 125 circRNAs, 1,911 mRNAs and 317 miRNAs between 96 and 0 h (*P* < 0.05, fold change > 2) (Tables S1–S4). The number of differentially expressed mRNAs and lncRNAs declined between 24 and 72 h. However, the number of differentially expressed mRNAs and lncRNAs increased sharply at 96 h (Figure 3B).

In addition, we searched the trVLP minigenome reads that included the Ebola leader region, VP40, GP, VP24, the Ebola trailer region and Renilla luciferase in a partial profile that was not mapped to the human genome. The number of reads increased between 0 and 48 h post infection and decreased between 48 and 96 h post infection, indicating viral replication and transcription primarily occur prior to 72 h (Figure 3C).

Based on the obtained set of differentially expressed genes and the viral replication curve, 0, 24, 48, and 72 h were selected as the time points for a series cluster analysis to identify key genes related to the effects induced by EBOV replication and transcription in human cells.

### Series cluster analysis

**Figure 4:**
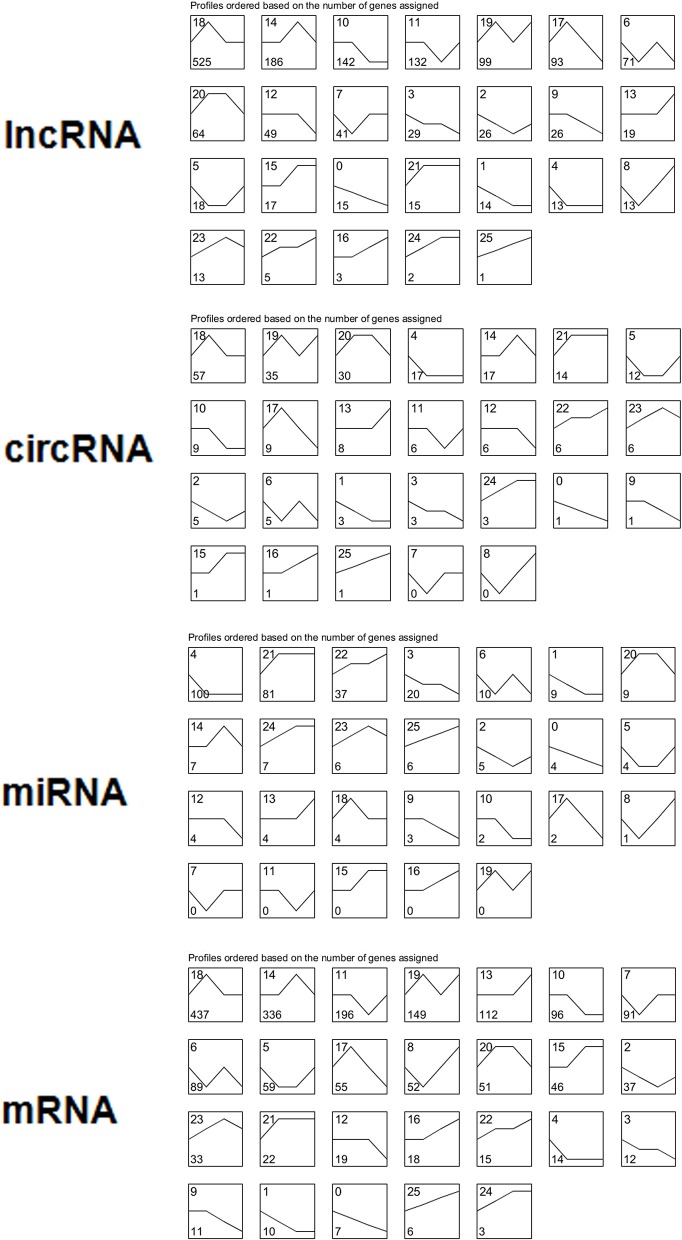
Overall expression patterns of lncRNAs, circRNAs, miRNAs, and mRNAs. All 26 expression profiles obtained for four types of RNAs were identified by performing a series cluster analysis. In each small graph, the number at the top left represents the expression type, and the number at the bottom left represents the number of genes in each expression type.

We performed a series cluster analysis and identified potential profiles representing the overall expression patterns of lncRNAs, miRNAs, circRNAs, and mRNAs. Of these, 497 down-regulated and 318 up-regulated lncRNAs, circRNAs, and mRNAs were selected to identify key genes throughout the viral life cycle (Figure 4 and Table S5).

It is important to characterize the relationship between the differential expression of these genes and viral replication. Therefore, a gene co-expression network analysis was performed to investigate the k-core value of each gene, which represents its co-expression ability.

### Gene co-expression network analysis

**Figure 5:**
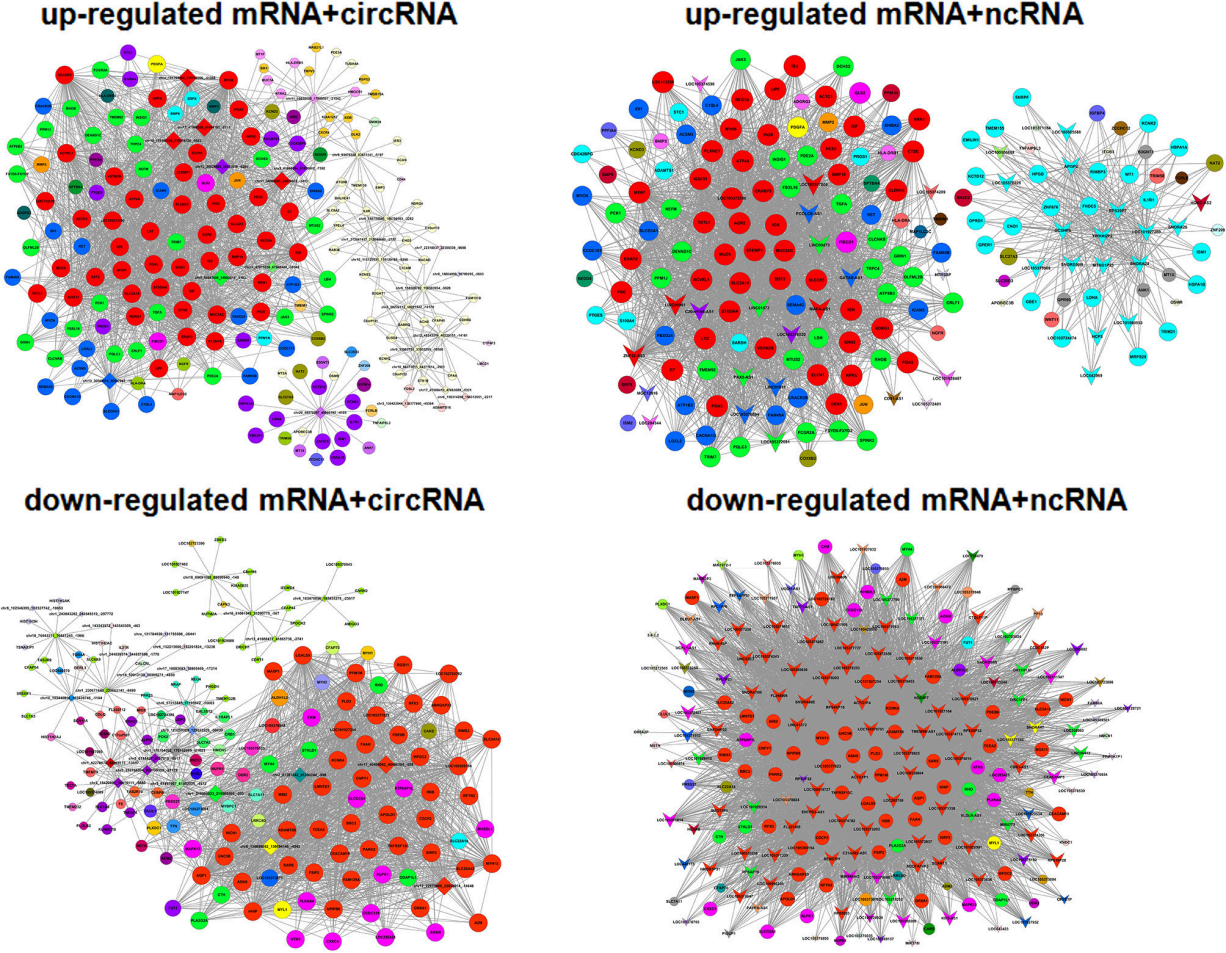
Gene co-expression networks. Up-regulated and down-regulated RNAs were selected to construct co-expression networks by calculating the Pearson correlation for each pair of RNAs. A round node represents a protein-coding gene, a triangular node represents a ncRNA, and a diamond node represents a circRNA. Red nodes represent the key regulatory genes with the highest k-core values. Node colors indicate k-core values. Node size represents the k-core power, and the distance between two nodes represents the interaction between the two RNAs.

Gene co-expression network analysis was performed to investigate key genes related to viral replication. We hypothesized that the genes with high k-core values may be involved in trVLP replication, modeling the EBOV life cycle. As shown previously, the selected genes were distributed into up-regulated and down-regulated groups, and a co-expression network analysis was performed for mRNAs and ceRNAs (lncRNA or circRNA) (Figure 5).

### Target prediction and ceRNA analysis

Competing endogenous RNA (ceRNA) analysis involved target prediction and the construction of a gene co-expression network between mRNA and ceRNA. Two ceRNA networks were established: one for circRNA-miRNA-mRNA interactions and another for ncRNA-miRNA-mRNA interactions (Tables S6, S7).

In this study, we used a trVLP system that models the life cycle of EBOV under BSL 2 conditions. This system represents a model for exploring how EBOV replication and transcription disturb normal cellular processes, such as DNA repair, intra-cellular anti-viral responses, energy production, membrane upkeep, and cytoskeletal changes. Although 293T cells are immortalized adrenal cells, these processes should be relatively unchanged between different cells and cell lines.

**Figure 6:**
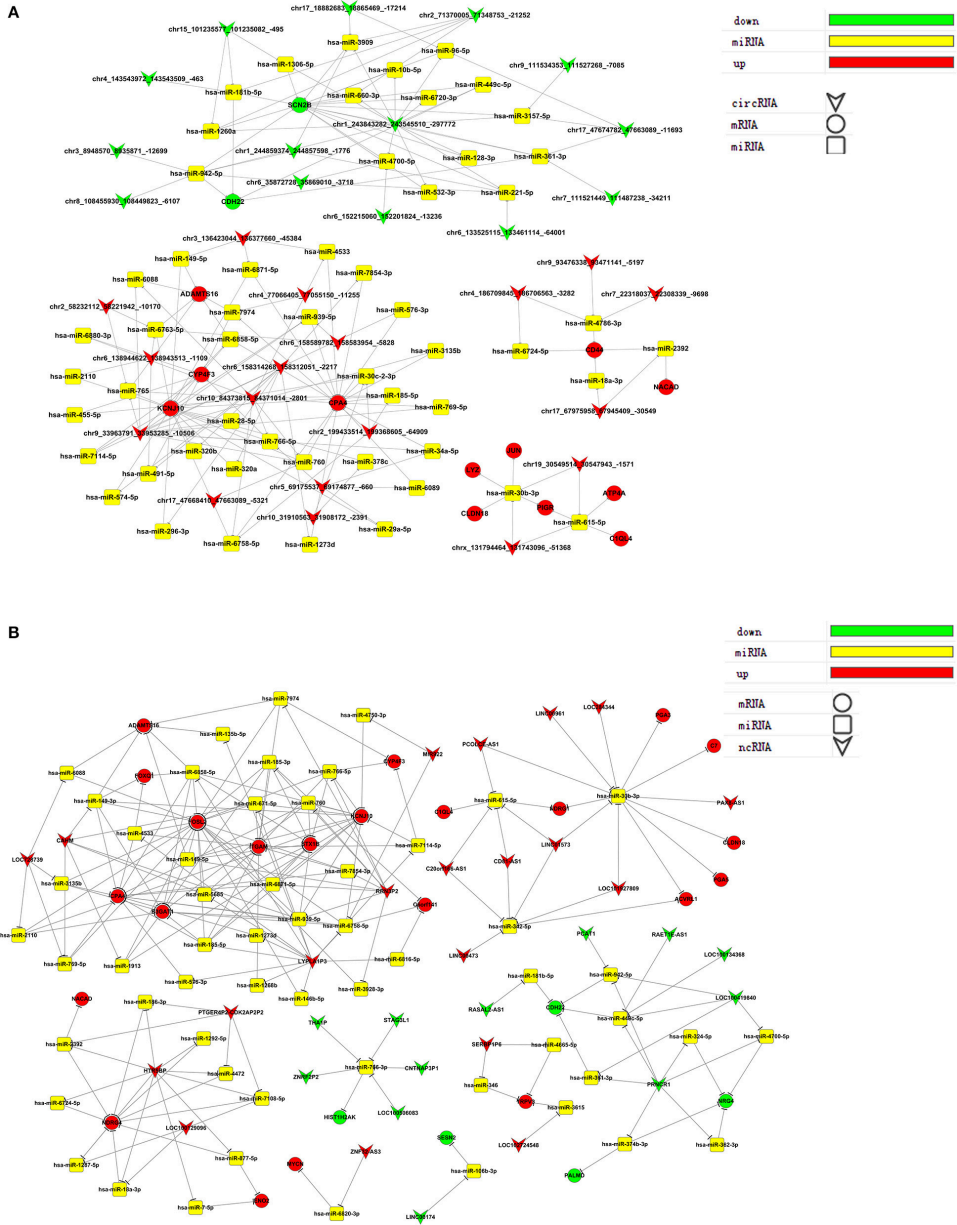
ceRNA networks. **(A)** circRNA-miRNA-mRNA and **(B)** lncRNA-miRNA-mRNA ceRNA networks.

We selected ceRNAs with K-Core values higher than 17 (circRNA) or 40 (lncRNA) to visualize networks to directly show the interactions between ceRNAs (Figure 6). From the ceRNA networks, we identified miR-30b-3p as an important potential target for many genes that were significantly up- or down-regulated post virus infection.

### Validation of gene expression associated with ceRNA-chr19-targeting microRNA

In gene co-expression analysis, we compared similarities in gene expression and analyzed the possible interactions of gene products to help reveal key genes in the interaction network. As a standard to select key genes for validation, we hypothesized that genes with high k-core values (as determined in the co-expression analysis) are involved in trVLP replication. Genes related to miR-30b-3p had the highest k-core values. Thus, we selected miR-30b-3p to validate its interaction details. As shown in (Figure 6), miR-30b-3p is a potential target for CLDN18 and may also be regulated by ceRNA-chr19. The gene of circRNA-chr19 is located at chr19: 30547942-30549514, and ZNF536 is its associated gene symbol. The length of circRNA-chr19 is 1572 bp.

**Figure 7:**
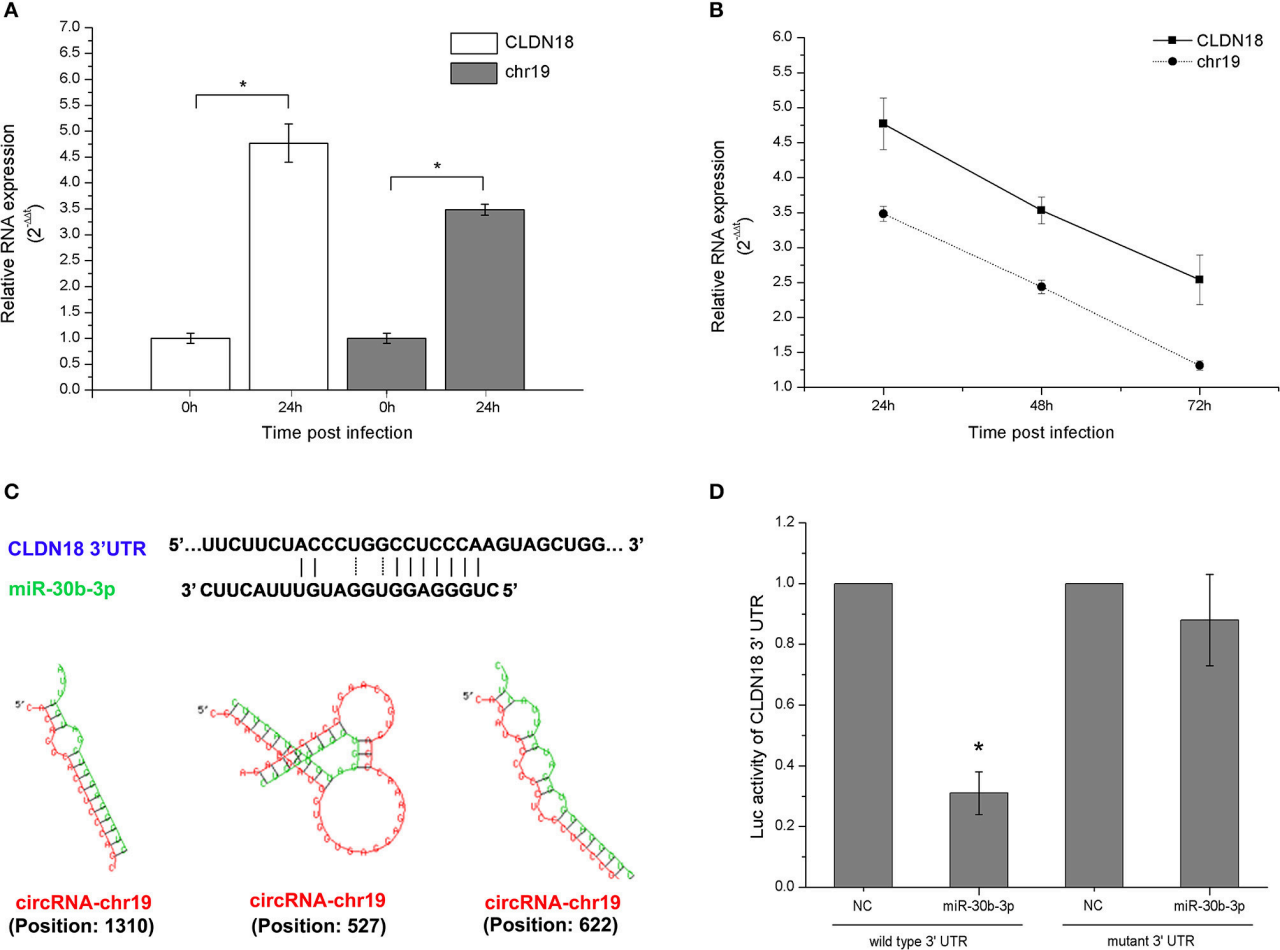
Gene expression changes post virus infection and transfection of circRNA-chr19-targeting microRNAs. **(A)** CircRNA-chr19 expression in 293T cells was analyzed via qPCR at 24 h and 0 h post virus infection and compared. **(B)** The expression levels of circRNA-chr19 and CLDN18 in 293T cells infected by virus. RNA expression, which was normalized by the expression level of GAPDH, is represented by the 2^−ΔΔ*t*^ value. **(C)** MiR-30b-3p matches the 3′-UTRs of CLDN18 and circRNA-chr19 (indicated by solid lines). The interactions of circRNAs and mRNAs with miRNAs were predicted by miRNA target prediction software, such as TargetScan and Bielefeld Bioinformatics Service online resource. **(D)** Wild-type and mutant CLDN18 3′-UTR activity were analyzed by luciferase reporter assays. The vector expressing miR-30b-3p was co-transfected with the vector containing the wild-type or mutant 3′-UTR. The results are presented as the mean ± SD of 3 different preparations, ^*^P < 0.05.

The gene expression detection results showed that both ceRNA-chr19 and CLDN18 were up-regulated post virus infection (Figure 7A), and circRNA-chr19 expression paralleled CLDN18 expression under the stimulation of virus reproduction (Figure 7B). Informatics analysis revealed that miR-30b-3p matches the 3′-UTR of CLDN18 and that there are also miR-30b-3p binding sites in the sequence of ceRNA-chr19 (Figure 7C). We constructed the miR-Report luciferase reporter to determine whether miR-30b-3p directly targets the 3′-UTR of CLDN18. The miR-Report luciferase reporter was co-transfected with miR-30b-3p expression plasmid. The luciferase activity of the reporter containing the wild-type 3′-UTR of CLDN18 was suppressed by miR-30b-3p, whereas the luciferase activity of the reporter containing the mutant 3′-UTR was not affected (Figure 7D).

**Figure 8:**
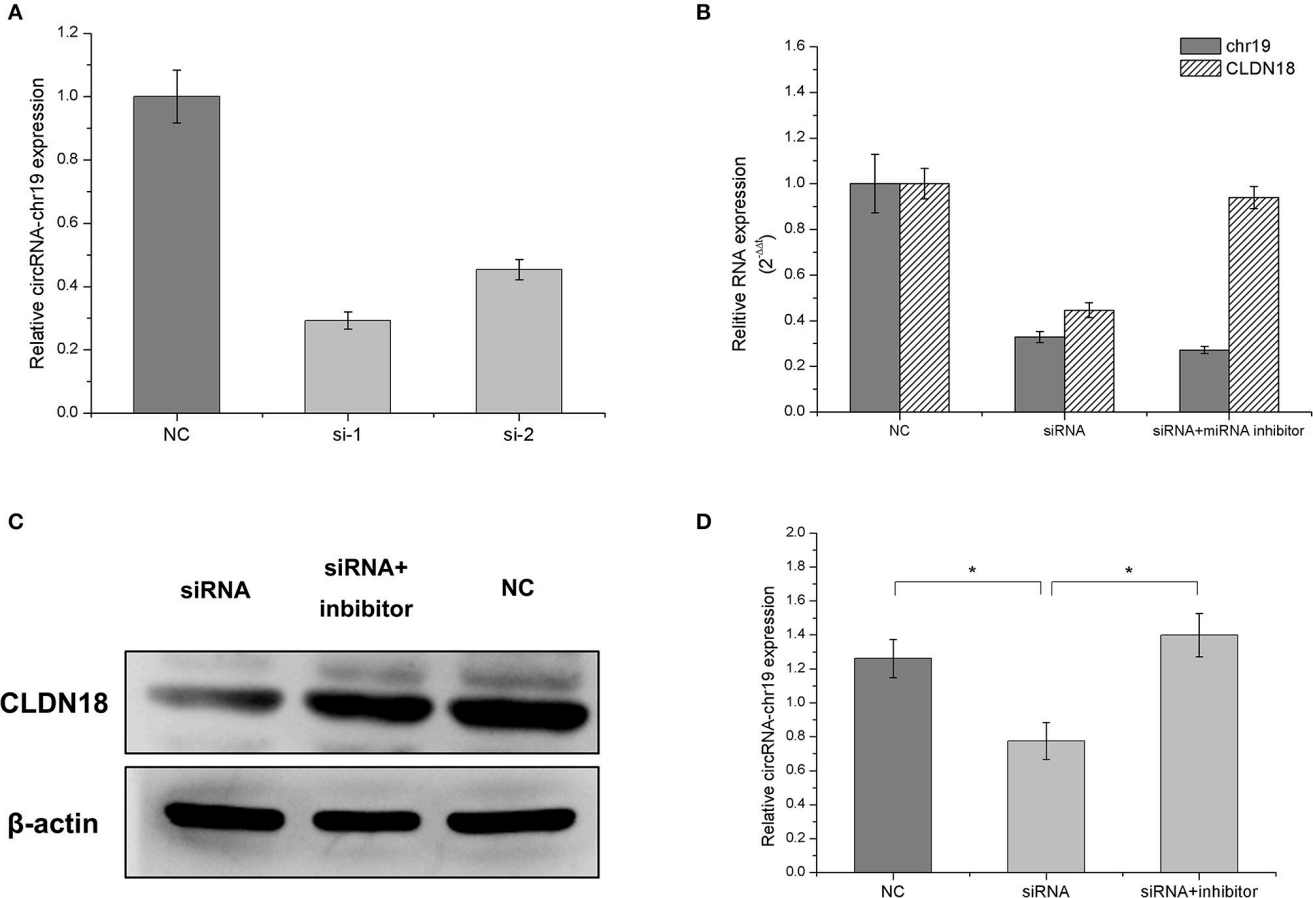
CircRNA-chr19 regulates CLDN18 expression via microRNAs. **(A)** The best inhibitory effect of small interfering RNAs (siRNAs) against circRNA-chr19 was achieved by si-1, which was analyzed by quantitative polymerase chain reaction (qPCR). GAPDH was used as a control. **(B)** The regulatory effect of knockdown of circRNA-chr19 by si-1 was destroyed by co-transfection with si-1 and an miR-30b-3p inhibitor. GAPDH was used as a control. **(C)** The protein expression levels of CLDN18 were analyzed by western blotting. β-Actin was used as a control. **(D)** The intensities of the respective images in **(C)** were quantified using Image J software. The results are represented as the mean ± SD of 3 different preparations, ^*^P < 0.05.

To determine how circRNA-chr19 regulates the expression of CLDN18, the knockdown effect of circRNA-chr19 was examined. The siRNA used in this detection was specific for circRNA-chr19, and the expression of ceRNA-chr19 was inhibited by si-1 and si-2 (Figure 8A). In addition, CLDN18 repression caused by circRNA-chr19 knockdown (using si-1) was reversed by the miR-30b-3p inhibitor (Figure 8B). This reversal was also confirmed at the protein level by western blot (Figures 8C,D).

## Discussion

Pathogens, particularly EBOV, which causes severe hemorrhagic fever, interact with host cells in many different ways (Zhao et al., 2016). In the past few decades, researchers have primarily focused on microRNAs that may be novel targets for Ebola treatment (Teng et al., 2015). However, there are many other types of RNAs that may play important roles in the viral replication and transcription process in host cells. For this reason, we conducted a genome-wide search for ceRNAs that may be responsible for the effects induced by EBOV infection and replication in human cells.

In this study, we used the trVLP system to complete a ceRNA network analysis under BSL 2 conditions. This system models the life cycle of EBOV under BSL 2 conditions and thus holds potential for understanding how EBOV replication and transcription disturb normal cellular processes. Additionally, this system is more convenient and may be implemented more safely to produce virus, infect cells, isolate samples and perform RNA extraction for sequencing. There are three main limitations of the trVLP system: First, in contrast to EBOV, the reproduction of trVLPs is dependent on the transfection efficiency of the target cells. Although Vero and BHK cells are susceptible cell lines, 293T cells are used for the production and passage of these trVLPs because of their high transfection efficiency. The expression of Tim-1 enhances trVLP infection in 293T cells by approximately 100-fold, and this supplementary process is important for the serial passage of trVLPs. Second, because the viral nucleocapsid proteins must be expressed in the target cells, this system cannot be used to assess the primary transcription of viruses. However, this system can still model most of the virus life cycle. Third, VP40, GP1,2, and VP24 can be expressed from the minigenome of the trVLPs. However, their position in the minigenome differs from that in the viral genome, which could influence their relative expression levels. Despite these limitations, the trVLP system can be used for studies of viral genome replication and transcription, morphogenesis and budding, attachment and entry into target cells over multiple infectious cycles under BSL 2 conditions (Hoenen et al., 2014). In 2016, a rapid screening assay of effective viral polymerase inhibitors of EBOV was performed using the trVLP system, and the assay results were confirmed using fully infectious Zaire EBOV (McCarthy et al., 2016). Given the high levels of virus replication and transcription, the lncRNAs and circRNAs that directly or indirectly interact with the virus could be easily and convincingly identified.

The mRNA, miRNA, lncRNA, and circRNA expression profiles were constructed by performing a series cluster analysis to identify RNA expression patterns, and up- or down-regulated RNAs were selected for further analysis to investigate viral replication and transcription patterns. Based on the gene co-expression network, a co-expression pattern was calculated for each selected RNA, reflected in the k-core values. The genes found in this study may be useful to further evaluate their functions during EBOV replication and transcription.

CircRNA and lncRNA are called “sponges,” and they indirectly regulate gene expression by interacting with microRNAs that regulate the expression of corresponding genes (Tay et al., 2014). Elucidating the functional ceRNAs involved in EBOV replication in host cells could be important for understanding Ebola pathogenesis and exploring potential therapeutic targets. In this study, we systematically produced the first ceRNA networks for EBOV infection, replication and transcription, which included lncRNAs, circRNAs, miRNAs, and mRNAs. Further refinement of these ceRNA networks was performed mainly by luciferase reporter analysis and gene knockdown effect analysis, which focused on the interactions between ceRNAs and their target RNAs. In the validation of the ceRNA interactions, we identified miR-30b-3p as a potential target of CLDN18 that might also be regulated by ceRNA-chr19. The first step in EBOV infection is to disable the immune system and then disable the vascular system, resulting in hemorrhagic symptoms that include blood leakage, a drop in blood pressure and hypotension. EBOV infection can also affect the liver, adrenal glands and gastrointestinal tract (leading to diarrhea) (Ansari, 2014). CLDN18, which is expressed in the human gastrointestinal tract, is a key gene in the development of inflammatory bowel disease (Lameris et al., 2013). Studies have shown that CLDN18 participates in epithelial disintegration, and epithelial cell lines expressing CLDN18-ARHGAP26 display a dramatic loss of epithelial phenotype (Yao et al., 2015). Claudins can also play important roles in transcellular and paracellular transport coupling (Gunzel, 2017). These studies related to CLDN18 indicate that CLDN18 has potential roles in cell permeability. However, whether claudins contribute to the vascular permeability and electrolyte disturbance in Ebola patients is not known. Although the purpose of this article was to perform a global screen of ceRNAs responsible for the effects induced by EBOV infection and replication, our study does provide a new perspective for interpreting the Ebola hemorrhagic symptom.

The purpose of this study was to establish ceRNA networks including ceRNAs responsible for the effects induced by EBOV replication and transcription. Many prediction results were obtained in this study. Interestingly, the discovery of circRNA-chr19 as a ceRNA for CLDN18 could increase the reliability of the prediction results, including ceRNA prediction and network construction, allowing them to be used as important tools to further explore potential targets and therapeutic agents in Ebola therapy.

The ceRNAs we predicted in this paper are important for further studies investigating how EBOV replication and transcription affect cellular mechanisms. Many methods to knock down ceRNAs, such as siRNA and CRISPR/Cas9, may be explored as therapeutic agents in Ebola therapy. These new therapeutic strategies targeting the ceRNAs involved in EBOV reproduction processes may play more important roles in the future.

## Additional information

All data has been submitted to GEO of NCBI and the series record GSE105414 provides access to all of the raw sequencing data.

## Author contributions

Z-YW, Lin-NL, and JQ conceived and designed the experiments. Z-YW and Z-DG wrote the manuscript. J-ML, Y-YF, Z-ZZ, YZ, Li-NL, and C-MZ performed some of the experiments.

### Conflict of interest statement

The authors declare that the research was conducted in the absence of any commercial or financial relationships that could be construed as a potential conflict of interest. The handling Editor declared a shared affiliation, though no other collaboration, with several of the authors: Z-YW, Z-DG, Z-ZZ, Y-YF, C-MZ, YZ, JQ, and Lin-NL.

## Supplementary material

Table S1 Differentially expressed non-coding RNAs.

Table S2 Differentially expressed circular RNAs.

Table S3 Differentially expressed messenger RNAs.

Table S4 Differentially expressed micro RNAs.

Table S5 Choosen genes for co-expression network and ceRNA analysis.

Table S6 ceRNA network (circRNA).

Table S7 ceRNA network (ncRNA).
